# Active Player Detection in Handball Scenes Based on Activity Measures

**DOI:** 10.3390/s20051475

**Published:** 2020-03-08

**Authors:** Miran Pobar, Marina Ivasic-Kos

**Affiliations:** Department of Informatics University of Rijeka, Rijeka 51000, Croatia; mpobar@uniri.hr

**Keywords:** object detector, object tracking, activity measure, Yolo, deep sort, Hungarian algorithm, optical flows, spatiotemporal interest points, sports scene

## Abstract

In team sports training scenes, it is common to have many players on the court, each with his own ball performing different actions. Our goal is to detect all players in the handball court and determine the most active player who performs the given handball technique. This is a very challenging task, for which, apart from an accurate object detector, which is able to deal with complex cluttered scenes, additional information is needed to determine the active player. We propose an active player detection method that combines the Yolo object detector, activity measures, and tracking methods to detect and track active players in time. Different ways of computing player activity were considered and three activity measures are proposed based on optical flow, spatiotemporal interest points, and convolutional neural networks. For tracking, we consider the use of the Hungarian assignment algorithm and the more complex Deep SORT tracker that uses additional visual appearance features to assist the assignment process. We have proposed the evaluation measure to evaluate the performance of the proposed active player detection method. The method is successfully tested on a custom handball video dataset that was acquired in the wild and on basketball video sequences. The results are commented on and some of the typical cases and issues are shown.

## 1. Introduction

Many applications of computer vision, such as action recognition, security, surveillance, or image retrieval, depend on object detection in images or videos. The task of object detection is to find the instances of real-world objects, such as people, cars, or faces. Detecting an object includes determining the location of that object and predicting the class to which it belongs. Object location and object classification problems both pose individual challenges, and the choice of the right object detection method can depend on the problem that needs to be solved. 

In our case, object detection, or specifically, leading player detection, can be very helpful for the recognition of actions in scenes from the handball domain. Handball is a team sport played in the hall on a handball court. Two teams consisting of a goalkeeper and six players participate in the game. The goal of the game is to score more points than the opposing team by throwing the ball into the goal of the opposing team, or by defending the goal, so that the opposing team does not score. The game is very fast and dynamic, with each team trying to arrange the action to defeat the defense and score a goal or to prevent the attack and defend the goal. All of the players can move all over the court except in the space 6 m in front of both goals where the goalkeepers are. The players move fast, change positions, and combine different techniques and handball actions, depending on their current role, which might be attack or defense. A player can shoot the ball toward the goal, dribble it, or pass it to a teammate during the attack, or block the ball or a player when playing defense. 

Different techniques and actions that players perform, as well as the frequent changes of position and occlusions, cause the shapes and appearances of players to change significantly during the game, which makes the detection and tracking process more challenging. The indoor court environment with relatively reflective floors is often illuminated with harsh lighting so that the shadows and reflections of players are usually present on the recordings and, due to the speed of action performance, motion blur occurs, which further complicates the tracking and detection problems. Additionally, the positions of the player and the distance to the fixed-mounted camera are constantly changing, so that players can be close to the camera, covering most of the image, or at a distance from the camera and occupy just a few pixels. The ball is also an important object for recognizing the handball scene, but it is even more complex to detect and track than the player, because it is even smaller, moves quickly, in different directions, and it is often covered by the player’s body or not visible because it is in the player’s hands [1].

**Figure 1:**
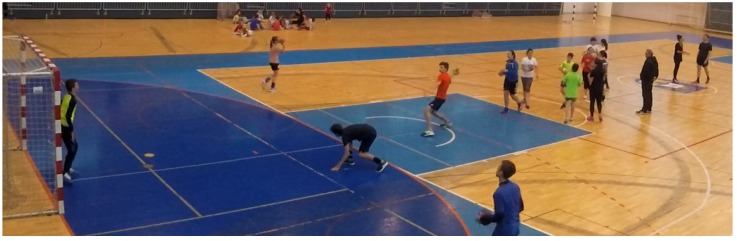
A typical training situation. The goalkeeper and the player in the center are performing the current task, while the rest are waiting, returning to queue, collecting the ball, etc.

Handball rules are well-defined and prescribe permitted actions and techniques, but during training, when adopting and practicing different techniques or game elements, the coach changes the rules to maintain a high level of player activity and a lot of repetition. Players then preform several sets of exercises in a row to train and improve certain techniques or actions, and to learn to position themselves properly in situations that imitate real game situations. In training sessions, each player also usually has his own ball, to reduce the waiting time to practice techniques and to train handball skills as quickly as possible. For example, a shooting technique involves a player that shoots the ball towards the goal and the goalkeeper that moves to defend the goal. Other players are waiting their turn to perform this activity or they gather their balls on the playground and then run to their position in the queue, as shown in Figure 1. 

Although all of the players move and interact in a certain moment, not all players contribute to the action, such as passing the ball or shooting at the goal, which is the most relevant for some exercise or for the interpretation of a handball situation. Thus, the focus should be on the players that are responsible for the currently most relevant handball action, and whose label can then be used to mark the whole relevant part on the video. Here, these players are referred to as active players.

The goal of the proposed method is to determine which of the players that are present in the video sequences are active at a certain time of the game or at the training sessions, and who of them performs the requested action at a given moment.

Detecting the player who performs an action of interest is particularly demanding during training sessions, because there are more players on the field than allowed during a normal game, there is more than one ball, because each player has their own ball and certain actions take place in parallel to make the players get the most out of the time to adopt or perfect the technique. The given goal is too complex for simple methods for people segmentation, such as background subtraction or Chroma keying, so, for this purpose, we suggest an active player detection method that combines player detection and tracking with an activity measure to determine the leading player(s). 

For the player detection, we suggest the use of deep convolution neural networks (CNNs) that have proven to be successful in classification and detection tasks in real-world images. In this paper, we will use Yolo V3 [2] which achieves great accuracy when detecting objects in the images. 

We propose three activity measures to determine the level of activity of a particular player. The one, named DT+STIP, is based on density of spatiotemporal interest points (STIPs) that are detected within the area of a player, the second, DT+OF, is computationally simpler and based on optical flow (OF) motion field between consecutive video frames, and the third, DT+Y, uses convolutional neural network (CNN) for determining player activity and classifying active and inactive players.

We consider the use of Hungarian assignment algorithm based on the bounding box positions of detected players, and the more complex Deep SORT tracker [3] that uses additional visual appearance features to assist the assignment process to track the detected players along the time dimension.

We have defined the evaluation measure for evaluate the performance of the proposed active player detection method. The proposed method is tested on videos that were taken in the gym during several training sessions, in real, uncontrolled indoor environment with cluttered background, complex light conditions and shadows, and with multiple players, on average 12, who practice and repeat different handball actions and dynamically change positions and occlude each other.

The rest of the paper is organized, as follows: in Section 2 we will briefly review the object action localization, sports player tracking and salient object detection. In Section 3, the proposed method that combines the Yolo object detector, three proposed activity measures and tracking methods to determine the most active player in sports scenes is described. We have defined the evaluation measure and applied the proposed method on a custom dataset that consists of handball scenes recorded during handball training lessons. The performance evaluation of the proposed active player detection method and comparison of different method setups with respect to the three proposed activity measures and two player tracking methods are given and discussed in Section 4 The paper ends with a conclusion and the proposal for future research.

## 2. Related Work

The goal of detecting the active or leading player in a sports video is to find the sequence of bounding boxes that correspond to the player currently performing the most relevant action. The task can be separated into player detection and player tracking, which produces sequences of bounding boxes that correspond to individual players, combined with saliency detection to determine which of the sequences belong to the active player. Alternatively, the first part of the problem can be viewed as a task of action localization in general, where the goal is to find the sequences of bounding boxes in videos that likely contain actions, not necessarily by explicitly trying to detect players, but possibly using other cues, such as only the foreground motion. 

Visual object tracking, including player tracking, is a very active research area that attracts dozens of papers to computer vision conferences annually [4], and numerous approaches have been developed for both the problem of multiple object tracking in general [5] and specifically for player tracking in sports videos. For the sports domain, player tracking is usually considered together with detection, e.g. in [6] for the case of hockey, [7] for handball, [8,9] for indoor, and [10,11,12,13] for outdoor soccer. These methods commonly attempt to exploit the specific knowledge regarding the domain of the given sport and about the video conditions, such as the distribution of the colors of the playing field or players to isolate potential areas that contain players [6,7,8,11,12,13], or the layout of the playing field to recover depth information [8,9,10,11]. Player detection ranges from template matching with handcrafted features [7] to machine learning approaches using e.g., a SVM classifier [13] or Adaboost [12], often with particle filter based tracking [6,9,13].

Recently, deep learning-based approaches to player detection, e.g. [14], are becoming increasingly attractive due to the increased detection performance as well as the reduced need for domain-specific knowledge. With the increased performance of convolutional neural network-based object detectors, relatively simple tracking-by-detection schemes that use the Hungarian algorithm to assign the detected bounding boxes to tracks only based on box dimensions have achieved good performance in the multiple object tracking task [15], such as tracking the leading player [16]. This work uses a similar approach. 

Determining who is the leading player among the detected players is related to the problem of salient object detection, where the aim is to detect and segment the object in an image that visually stands out from its surroundings, which would be in the center of attention in the human system. In addition to finding naturally (visually) distinct regions (bottom-up-saliency), the criteria for saliency may be based on a specific task, expectations, prior knowledge, etc. (top-down saliency) [17]. In this sense, finding the active player among the detected players can be regarded as a top-down saliency task. Different cues have been used for saliency detection, such as those that are based on intensity or color contrast, informed by the sensitivity of human perception to color, or those that are based on location information, e.g., treating center region of an image as more likely to contain salient objects [18]. With the popularization of deep learning models for object detection, similar models are also increasingly used for salient object detection [19]. In the video context, motion-based saliency cues can be used, e.g., based on by directional masks applied to the image [18] or on optical flow estimation [20,21]. In this work, motion cues that are based on optical flow and STIPS are used to inform saliency of a player. A survey of salient object detection work can be found in [17,19].

In the area of action localization, the goal is to find the sequence of bounding boxes in a sequence of video frames that correspond to an action. Generating a set of candidate spatiotemporal regions, followed by the classification of the candidate region into an action class or the non-action class, is commonly utilized to perform this. Jain et al. [22] first segment a video sequence into supervoxels, and then use a notion of independent motion evidence to find the supervoxels where motion deviates from the background motion, which signals the potential area of interest where action is likely going to be detected. Similarly, in [23], a motion saliency measure that is based on optical flow is used along with image region proposal based on selective search to select candidate spatiotemporal regions in videos with actions and to disregard areas with little movement where no action is likely. Kläser et al. [24] explicitly split the action localization task into first detecting the persons in video while using a HOG [25] descriptor and a sliding window support vector machine classifier and then tracking the detected persons.

In [26], basketball players are detected and tracked in the video for the task of event detection, and the most relevant player is implicitly learned from the correspondence of video frames to the event label in a LSTM model trained to classify these events.

## 3. Proposed Method for Active Player Detection

**Figure 2:**
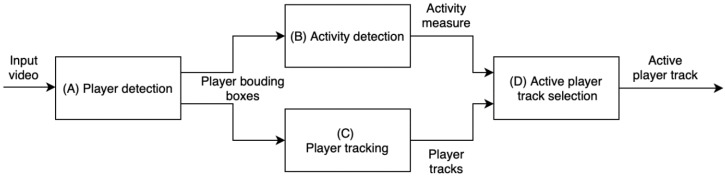
An overview of the active player track detection. Player detector provides bounding boxes for each input video frame (**A**). Player activity measure is computed for each detected player bounding box in each frame (**B**) and players are tracked across frames (**C**). Combining the track information from (C) and activity measure from (B), final decision about the active player track is made (**D**).

The goal of the proposed method is to automatically determine the player or players in the scene that are responsible for the current action, among all of the players present in the scene. Figure 2 presents the overview of the proposed method for active player detection.

First, the players are detected in the input video while using a CNN-based object detector. Here, the YOLOv3 detector pre-trained on the MS-COCO dataset was used to mark the players on each frame of the video sequence with their bounding boxes (Figure 2A), using the detector’s general person class. In this case, the object detector does not distinguish between active and inactive players, i.e. those who perform the action of interest from those who do something else. The activity measures for each detected player in a frame are computed based on motion features that are extracted within each player’s bounding box in that video frame (Figure 2B). To determine a player’s overall activity in a time period, it is necessary to know their activity measure in each frame, but to also uniquely identify that player throughout the period of interest, i.e., to track them through the sequence. While considering that the object detector only determines the class of a detected object (e.g., person), without distinguishing between objects of the same class, the tracking of player’s identities between frames is completed outside the detection step (Figure 2C). Finally, the information about player activity in each frame and the track information is joined to determine the track of the most active or leading player in the whole video (Figure 2D). 

Each of the steps is described in more detail below.

The Hungarian assignment algorithm was used to track the players, with a distance function based on properties of bounding boxes as the baseline method. As an alternative, the Deep SORT [3] method, which uses additional visual features to aid the tracking, is considered. Since Deep SORT gave better tracking results, only this method was used when testing the activity measures.

Different ways of computing player activity were considered and three activity measures are proposed. The first measure, named DT+OF, is based on the optical flow calculated within a player’s bounding box and considers the velocity component of the motion, while the second measure, named DT+STIP, uses the density of the spatiotemporal interest points (STIPs) within the bounding boxes to determine the activity measure. The third method, called DT+Y, uses the YOLOv3 network to classify a player into an active or inactive class.

The input videos used as input data were recorded during the handball training involving 15 and more players. Each video sequence was selected and trimmed, so that it contains all the steps of the given action.

### 3.1. Player Detection

The goal of object detectors is to locate and classify objects on the scene. The detected objects are typically marked with a bounding box and labeled with corresponding class labels.

The current focus in object detection is on convolutional neural networks (CNNs) that were, at first, used for image classification, but have later been extended to be able to both detect and localize individual objects on the scene. This can be achieved by the independent processing of image parts that are isolated while using a sliding window that is moved over the image [27]. Multiple scales of windows are used in order to take into account different sizes of objects in the image. If the classifier recognizes the contents of the window as an object, the window is used as the bounding box of the object and the corresponding class label is used to label the window. After processing the entire image, the result is a set of bounding boxes and corresponding class labels. As a certain object can be partially or fully contained within multiple sliding windows, a large number of duplicate predictions can be generated, which can then be rejected while using non-maximum suppression and a confidence threshold. Since the sliding window approach essentially makes an image classification for each window position, a naive implementation can be much slower and computationally expensive when compared to simple image classification.

Here, the role of the object is to detect the players in each frame of the handball videos. Ideally, the detector should be as precise as possible, yet less computationally demanding, so that in can detect objects in real time. It has been previously shown [28,29] that Mask R-CNN [30] and Yolo have comparable performances of player detection in handball scenes, but, since Yolo was significantly faster and was successful for player detection in previous work [31], it is used for this experiment. The YOLOv3 detector was used here using the pre-trained parameters on the COCO dataset with the standard Resnet-101-FPN network configuration, with no additional training.

YOLOv3 is the third iteration of the YOLO object detector [32], which performs a single pass through a neural network for both detecting the potential regions in the image where certain objects are present, and for classifying those regions into object classes. The object detection task is framed as a problem of regression from image pixels to objects’ bounding box coordinates and associated class probabilities. 

The architecture of the YOLOv3 detector is a convolutional network that consists of 53 convolutional layers of 3 × 3 and 1 × 1 filters with shortcut connections between layers (residual blocks) is used for feature extraction. The last convolutional layer of the network predicts the bounding boxes, the confidence scores, and the prediction class. YoloV3 predicts candidate bounding boxes at three different scales using a structure that is similar to feature pyramid networks, so three sets of boxes are predicted at each feature map cell for each scale, to improve the detection of objects that may appear at different sizes.

**Figure 3:**
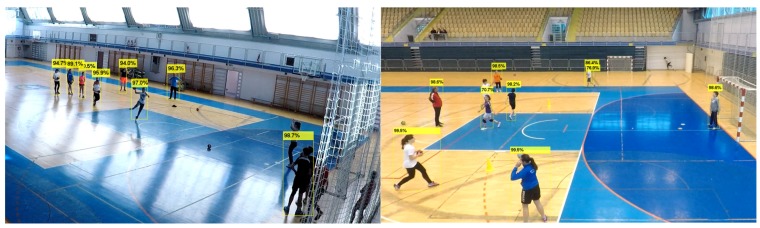
Bounding boxes with confidence values as results of person detection with YOLOv3.

For the task of active player detection, only the bounding boxes and confidence values for the objects of the class "person" were used. An experimentally determined confidence threshold value of 0.55 was used to filter out the unwanted detections and obtain good balance of high detection and low false positives rates. Figure 3 shows an example of detection results for the "person" class.

### 3.2. Player Tracking

The detections that were obtained with the Yolo detector are independent of each processed frame and, thus, contain no information about which bounding boxes correspond to the same objects in consecutive frames. Finding this correspondence between bounding boxes and player identities across frames is required to obtain the player trajectories. An additional post-processing step for player tracking is used for this purpose. Two methods are considered for this task. The first is a simpler approach utilizing the Hungarian algorithm, which assigns player bounding boxes detected in different frames into the player track while taking the dimensions and positions of the bounding box into account. In the second approach, the Deep SORT method [3] is used, which uses additional visual features to determine which detection in a frame matches a previously detected player. 

#### 3.2.1. Tracking Using the Hungarian Algorithm

In the first video frame, the player tracks are initialized, so that a track ID is assigned to each detected player bounding box. In the next frames, the individual detected bounding boxes are assigned to previously initialized tracks while using the Munkres’ version of the Hungarian algorithm [33], whose objective is to minimize of the total cost of assigning the detections to tracks. Assigning a bounding box to a track has a cost value that depends on the scale difference and the relative position of the candidate bounding box and the box that was already assigned to the track in the previous frame to take simplified visual and spatial distances into account.

More precisely, a new bounding box $B_{b}$ will be assigned to a player track $T_{b-1}\;$if it has the minimal cost computed as a sum of the linear combination of Euclidean distance between the centroids ($C_{b-1}$) of the last bounding box assigned to the player track $T_{b-1}\;$and the detected centroids ($C_{b}$) and the absolute area difference of the detected bounding boxes area ($P_{b}$) and the last bounding box area ($P_{b-1}$) assigned to the track $T_{b-1}\;$(1):

(1) $$B_{b}(C_{b},P_{b}):\;b=\underset{i}{\mathrm{argmin}}\sum_{i\in F}(wd_{2}\left(C_{b-1},C_{i}\right)+\left(1-w\right)\left|P_{i}-P_{b-1}\right|);w\in \left[0,1\right];\;d_{2}\left(C_{b-1},C_{i}\right)<T.$$

where the bounding box $B_{b}(C_{b},P_{b})$ is represented with its centroids $C_{b}$ and the area $P_{b}$, T∈R is a distance threshold between centroids in consecutive frames, w is the adjustable parameter that determines the relative influence of the displacement and the change of bounding boxes area in consecutive frames and F is the set of all the bounding boxes within the current frame.

The number of tracks can also change throughout the video, since players can enter or exit the camera field of view at any time. Additionally, the detection of players is not perfect, so some tracks should resume after a period where no detection was assigned. The distance threshold T is used to control the maximum allowed distance between a detected bounding box and a last bounding box assigned to the track. Any box whose distance to a track is greater than this threshold cannot be assigned to that track, even if it is the closest one to the track. If for M∈N consecutive frames no detections are assigned to a track, the track is considered to be completed and no further detections can be added to it. The values of M and T are experimentally determined and set to 20 and 100, respectively.

**Figure 4:**
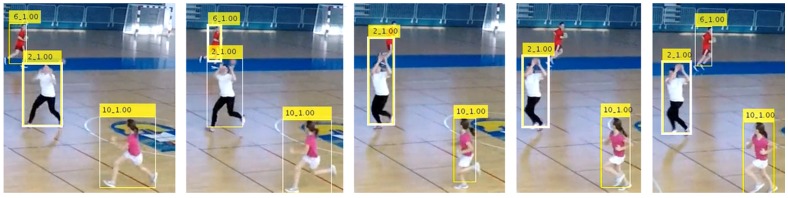
Player tracking across frames.

The Figure 4 shows an example of a sequence of frames from a video, with player bounding boxes being additionally associated with a track IDs across five different frames, such that, on different sequential frames, the same player has the same ID. It can be noted that the player with track ID 6 is not detected in several frames, but the tracking continues with the correct ID after he was detected again in the last shown frame.

#### 3.2.2. Tracking Algorithm—DeepSORT

DeepSORT [3] is a tracking algorithm that is based on the Hungarian algorithm that, in addition to the parameters of the detected bounding boxes, also considers the appearance information regarding the tracked objects to associate new detections with previously tracked objects. The appearance information should be useful, in particular, with re-identifying players that were occluded or have temporarily left the scene. As with the basic application of Hungarian algorithm, it is able to perform the tracking online, i.e. it does not need to process the whole video at once, but only needs to consider the information about the current and the previous frames to assign detections to tracks. 

Like in the previous case, in the first frame, a unique track ID is assigned to each bounding box that represents a player and has a confidence value higher than a set threshold and the Hungarian algorithm is used to assign the new detections to existing tracks, so that the assignment cost function reaches the global minimum. The cost function entails the spatial (Mahalanobis) distance $d^{\left(1\right)}$ of a detected bounding box from the position that is predicted from its previously known position, and a visual distance $d^{\left(2\right)}$ that compares the appearance of a detected object with a history of appearances of the tracked objects. The cost of associating a detected bounding box $B_{b}$ to player track $T_{b-1}$ that ends with the bounding box $B_{b-1}$ is given by the expression:

(2) $$c_{b,b-1}=\lambda d^{\left(1\right)}\left(B_{b-1},B_{i}\right)+\left(1-\lambda \right)d^{\left(2\right)}\left(B_{b-1},B_{b}\right),$$

where λ is a settable parameter that determines the relative influence of the spatial and the visual distances $d^{\left(1\right)}$ and $d^{\left(2\right)}$.

The distance $d^{\left(1\right)}$ is given by the expression:

(3) $$d^{\left(1\right)}\left(B_{b-1},B_{b}\right)={\left(d_{b}-y_{b-1}\right)}^{T}S_{b-1}^{-1}\left(d_{b}-y_{b-1}\right),$$

where $y_{b-1}$ and $S_{b-1}$ are the mean and the covariance matrix of the last bounding box observation assigned to player track $T_{b-1}$, and $d_{b}$ is the detected bounding box. 

The visual distance $d^{\left(2\right)}$ is given by the expression:

(4) $$d^{\left(2\right)}\left(B_{b-1},B_{b}\right)=\min\left\{1-r_{b}^{T}r_{k}^{\left(b-1\right)}|r_{k}^{\left(b-1\right)}\in T_{b-1}\right\},$$

where $r_{b}$ is the appearance descriptor obtained from the part of the image within the detected bounding box $B_{b}$ and $T_{b-1}\;$is the set of last 100 appearance descriptors $r_{k}^{\left(b-1\right)}$ that are associated with the $T_{b-1}\;$track.

The goal of the $d^{\left(2\right)}$ measure is to select the track where visually the most similar detection was previously found to the current detection. The similarity is computed via the cosine distance between the appearance descriptors of the $B_{b}\;$detected frame and $T_{b-1}$ track. The 128-element descriptors are extracted while using a wide residual neural network with two convolutional layers and six residual blocks that was pre-trained on a person re-identification dataset of more than one-million images of 1261 pedestrians. The appearance descriptor vectors are normalized to fit within a unit hypersphere so that the cosine distance can be used [3].

When a detection cannot be assigned to any track because it is too far from any track according to the distance $d^{\left(1\right)}$, or is not visually similar enough to any previous detection according to the distance $d^{\left(2\right)}$, a new track is created. The maximum allowed $d^{\left(1\right)}$ and $d^{\left(2\right)}$ distances when an assignment is still possible is a settable parameter. A new track is also created whenever there are more detected players in a frame than there are existing tracks. A track is abandoned if no assignment has been made to a track for M∈N consecutive frames, and a new track ID will be generated if the same object re-appears later in the video.

### 3.3. Activity Detection

The object detector provides the location of all the players present in the scene in bounding boxes, but it has no information regarding the movements or activities of the players. Some information about movement can be obtained by first tracking the players and then by calculating the shift of the bounding box centroid across frames. However, it is expected that strong and sudden changes in both velocity and appearance that cannot be described by the shift of the bounding box centroid alone will characterize various actions in sports videos. Three activity measures are proposed (DT+OF, DT+STIPS, and DT+Y) to better capture the information about the activity of the players, as noted earlier.

Both the optical flow estimation method and STIPs detection by themselves only consider sections of video frames without taking the object identities into account. Thus, the information about player locations obtained with Yolo is combined with the activity measure obtained from either optical flow or STIPs within the player’s bounding box to measure the individual player’s activity.

#### 3.3.1. Optical Flow-Based Activity Measure—DT+OF

In the proposed DT+OF activity measure, the optical flow estimate from time-varying image intensity is used to capture the information regarding speed and direction of movement of image patches within players bounding boxes $B_{b}$, which may correspond to player activity [21]. Movement of any point on the image plane produces a two-dimensional (2D) path $x\left(t\right)\equiv {\left(x\left(t\right),y\left(t\right)\right)}^{T}$ in camera-centered coordinates. The current direction is the velocity V=dx(t)/dt. The 2D velocities of all visible surface points make up the 2D motion field.

The optical flow motion field in consecutive video frames is estimated while using the Lucas–Kanade method [34]. In the Lucas–Kanade method, the original images are first divided into smaller sections, and it is assumed that the pixels within each section will have similar velocity vectors. The result is the vector field V of velocities, where, at each point (x, y), the magnitude of the vector represents the movement speed and its angle represents the movement direction.

**Figure 5:**
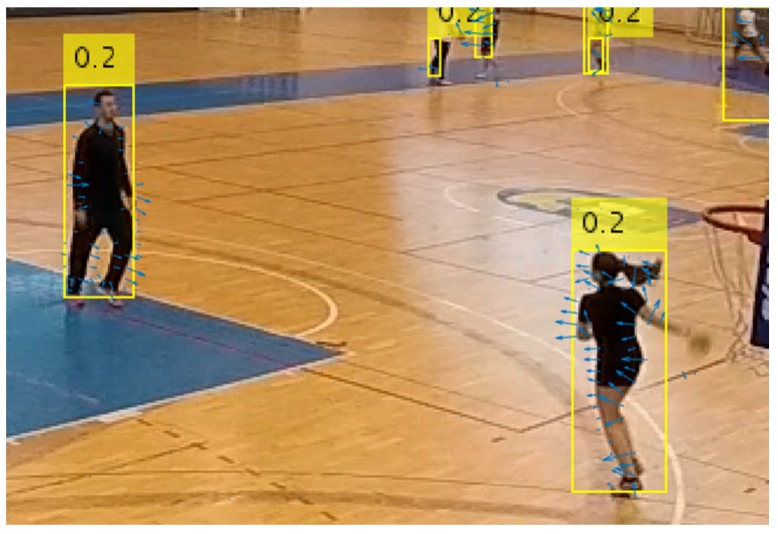
Player bounding box (yellow) and optical flow vectors (blue).

Figure 5 visualizes an example of the calculated optical flow vectors between correspondent bounding boxes in two video frames from the dataset. The direction and length of blue arrows represent the direction and magnitude of optical flow at each point. 

The activity measure ($A_{b}^{OF}$) of a player detected with its bounding box ($B_{b}$) is calculated in each frame as the maximum optical flow magnitude $V_{x,y}$ within the area $P_{b}$ of the bounding box:

(5) $$A_{b}^{OF}=\underset{B_{b}}{\max}\left|V_{x,y}\right|;\;x,y\;\mathrm{within}\;B_{b}.$$

The assumption is that the players performing a sports action make more sudden movements that will result in larger optical flow vectors within the player bounding boxes. The maximum value of the optical flow is used in order to make the comparison between players with different bounding box sizes simple.

#### 3.3.2. STIPs-Based Activity Measure—DT+STIP

The DT+STIP activity measure is based on spatiotemporal interest points (STIPs). STIPs are an extension of the idea of local points of interests in the spatial domain, i.e. of points with a significant local variation of image intensities into both spatial and temporal domains, by requiring that image values have large variation in both spatial and temporal directions around a point.

It is expected that the higher density of STIPs in a certain area will point to a region of higher movement, which might correspond to the higher level of player’s activity, since STIPs capture the spatiotemporal “interestingness" at different points in the image, and most actions in sports videos are characterized by strong variations in velocity and appearance over time [16]. Thus, an activity measure $A_{b}^{STIP}$ that is based on STIPs density is calculated for a player with a bounding box $B_{b}$ and area $P_{b}$. in a certain frame, as:

(6) $$A_{b}^{STIP}=\;\frac{\#STIP}{P_{b}};\;STIP\;\mathrm{within}\;B_{b}.$$

**Figure 6:**
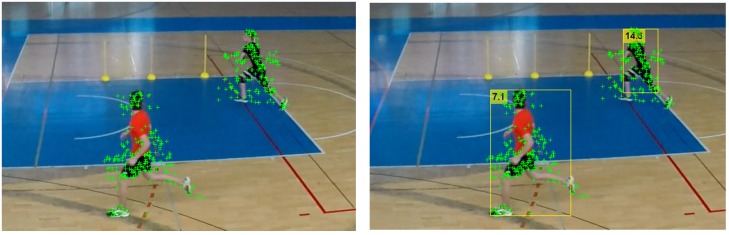
Fusion of bounding box and spatiotemporal interest points.

Figure 6 shows an example of detected STIPS in a frame of video, as well as the detected STIPs superimposed on the detected players’ bounding boxes.

A number of methods have been proposed to detect the STIPs. The method that is proposed in [35] is based on the detection of the spatiotemporal corners derived from Harris corner operator (Harris3D), in [36] on Dollar’s detector that uses a Gaussian filter in the space domain and a Gabor band-pass filter in the time domain and obtains a denser sampling by avoiding scale selection, in [37] on Hessian3D derived from SURF or selective STIPs detector [38] that focuses on detecting the STIPs that likely belong to persons and not to the possibly moving background. The Harris3D detector is used in the experiments described here.

In the Harris3D STIPs detector, a linear scale-space representation L is first computed by convolving the input signal f with an anisotropic Gaussian kernel g, i.e., a kernel with independent spatial and temporal variances [35]:

(7) $$L\left(.;\sigma^{2},\tau^{2}\right)=g\left(.;\sigma^{2},\tau^{2}\right)*f\left(.\right),$$

where $\sigma^{2}$ and $\tau^{2}$ represent the spatial and temporal variance, respectively, and the kernel g is defined as:

(8) $$g\left(x,y,t;\sigma^{2},\tau^{2}\right)=\frac{1}{\sqrt{{\left(2\pi \right)}^{3}\sigma^{4}\tau^{2}}}e^{\frac{-\left(x^{2}+y^{2}\right)}{2\sigma^{2}}-\frac{t^{2}}{2\tau^{2}}}.$$

A spatiotemporal second-moment matrix μ, which is composed of first order spatial and temporal derivatives of L averaged using a Gaussian weighting function g, is computed:

(9) $$\mu =\;g\left(.;\sigma_{i}^{2},\tau_{i}^{2}\right)*\left(\begin{array}{ccc} L_{x}^{2} & L_{x}L_{y} & L_{x}L_{t}\\ L_{x}L_{y} & L_{y}^{2} & L_{y}L_{t}\\ L_{x}L_{t} & L_{y}L_{t} & L_{t}^{2} \end{array}\right),$$

where $L_{x},L_{y}$, and $L_{t}$ are the first-order derivatives of g∗f in the x, y, and t directions.

The spatiotemporal interest points are obtained from the local maxima of the corner function H:

(10) $$H=\det\left(\mu \right)-k\cdot {\mathrm{trace}}^{3}\left(\mu \right),$$

where *k* is a parameter that was experimentally set to be k≈0.005 [35].

The STIPs are extracted while using the with default parameters from the whole video. 

#### 3.3.3. CNN-Based Activity Measure—DT+Y

The DT+Y activity measure uses the YOLOv3 to recognize the level of activity of detected player, or to determine whether the player is active or not. 

The YOLOv3 network that was, in the previous cases, only used for player detection, was additionally trained on custom data to classify the detected player as either active or inactive so that the class person was replaced with two classes: active player and inactive player. Aside from the number of detected classes, the architecture of the network is the same as the network used for player detection in DT+OF and DT+STIPS. 

The training was completed for 70,000 iterations on 3232 frames that were extracted from 61 videos in our training dataset. The frames were manually labeled either as active or as inactive player. The learning rate was 0.001, momentum 0.9, and decay was 0.0005. The input image size was 608 × 608 pixels without using YOLO’s multiscale training. Data augmentation in the form of random image saturation and exposure transformations, with maximum factors of 1.5, and with random hue shifts by, at most, 0.1 was used.

The activity measure $A_{b}^{Y}$ for each bounding box$\;B_{b}\;$corresponds to confidence value c of the active player class, such as:

(11) $$A_{b}^{Y}=\;c\left(active\;player\;class\left(B_{b}\right)\right).$$

Additionally, a threshold value can be defined for the classification accuracy of the active player class, above which the player will be considered active, and inactive otherwise.

### 3.4. Determining the Most Active Player

**Figure 7:**
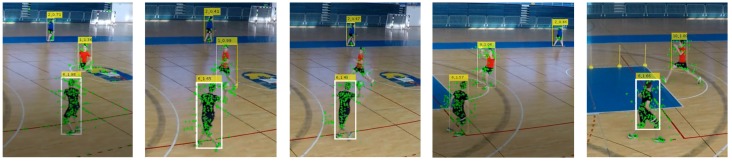
Indicating the most active player on each frame (marked with thick white bounding box).

Throughout the sequence, different players will have the highest activity scores at different times. The activity scores for each player in individual frames are first aggregated into the track scores to select the most active player in the whole sequence. In Figure 7, a thick white solid bounding box line indicates the most active player for each frame (here player with ID 6). As shown in the Figure 7, in the fourth frame, in the case where multiple players have the same activity level, none is marked as being active.

The track scores are represented by a vector whose elements represent the ranking by activity measure in a frame. For example, if at the first frame the track with ID 6 (track 6) has the highest activity measure, and track 1 has the second largest activity measure, the track score at frame 1 for track 6 will be 1, and for the track 2 it will be 2. Table 1 provides an example of the ranking of players in each individual frame by their activity level and determining the most active player in a given time frame. 

Finally, the active player’s track is the one that is most commonly ranked first by the activity level in the whole sequence, i.e. whose track score vector contains the most ones among all track score vectors. 

**Figure 8:**
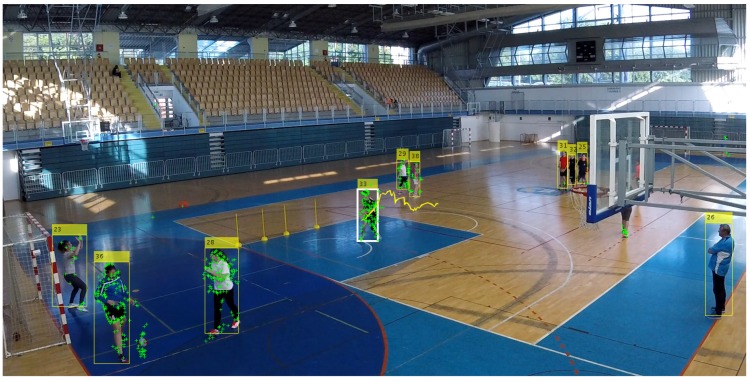
Detected most active player (white thick bounding box) and his trajectory through the whole sequence (yellow line).

The result of the proposed method for determining the most active player is a series of active player bounding box representations $B_{b}(C_{b},P_{b})$ that are stored in the player track $T_{b}=(B_{1},\;B_{1},\;\ldots .B_{b})$ from the beginning to the end of performing an action. The graphical representation of the centroids $C_{b}$ of each bounding box $B_{b}(C_{b},P_{b})$ through the motion sequence corresponds to the trajectory of his movement that is shown with the yellow line (Figure 8). 

**Figure 9:**
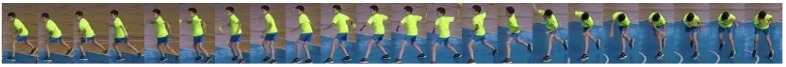
Extracted collage of the most active player’s action.

The active player track can ultimately be represented by a collage of thumbnails that correspond to the bounding boxes of the most active player, showing the stages of an action (Figure 9).

## 4. Performance Evaluation of Active Player Detection and Discussion

The proposed method was tested on a custom dataset that consists of scenes that were recorded during a handball training session. The handball training was organized in a sports hall or sometimes in an outdoor terrain, with a variable number of players without uniform jerseys, with challenging artificial lighting and strong sunshine, with cluttered background and other adverse conditions.

**Figure 10:**
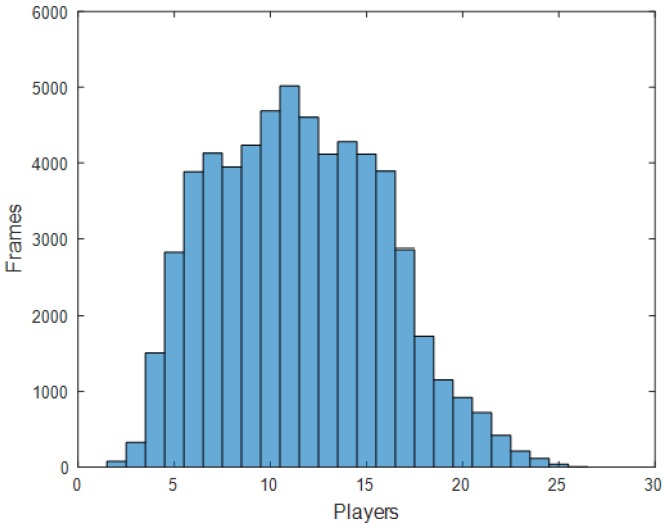
Distribution of number of players per frames.

The dataset consists of 751 videos, each containing one of the handball actions, such as passing, shooting, jump-shot, or dribbling. Multiple players, on average, 12 of them, appear in each video, as shown in Figure 10. Table 2 provides the statistics of the number of players per frame.

Each player can move in any direction, but mostly one player performs an action of interest, so he is considered as the active player. Therefore, each file is only marked with one action of interest. The scenes were shot with stationary GoPro cameras that were mounted on the left or right side of the playground, from different angles and in different lighting conditions (indoor and outdoor). In the internal scenes, the camera is mounted at a height of 3.5 m and in the outside scenes at 1.5 m. The videos were recorded in full HD (1920 × 1080) at 30 frames per second. The total duration of the recordings used for the experiment was 1990 s.

In the experiment, the individual steps of the proposed method and the method as a whole were tested, from person detection to selecting the trajectory of an active player.

**Figure 11:**
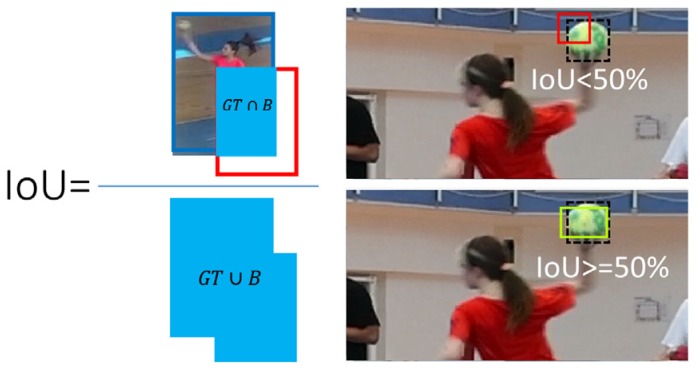
Visual representation of intersection over union (IoU) criteria equal to or greater than 50%.

For the player detection task, the Yolo detector was tested on full-resolution videos and without frame skipping. The performance of the Yolo detector was evaluated in terms of recall, precision, and F1 score [39]. The detections are considered to be true positive when the intersection over union of the detected bounding box and the ground truth box exceeded the threshold of 0.5. The intersection over union measure (IoU) is defined as the ratio of the intersection of the detected bounding box and the ground truth (GT) bounding box and their union, as in Figure 11. 

**Figure 12:**
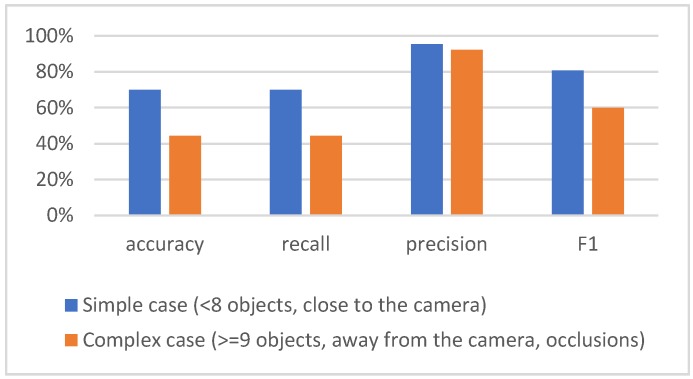
Results of player detection with Yolo in simple and complex scenarios.

The results of the detector greatly depend on the complexity of the scene, which in the used dataset depends on the number of players on the scene and their size, as well as on the distance from the camera and the number of occlusions. For example, if there are up to eight players on the scene, close to the camera, the results are significantly better than in the case when there are nine or more players on scene that are far from the camera and occlusions exist, as shown in Figure 12. The background is similar in both cases, and it includes the ground surface and its boundaries, the advertisements along the edge of the terrain, and chairs in the auditorium.

The obtained results confirm that player detection, which is the first step of the active player detection method, provides a good starting point for the next phases of the algorithm.

In the next step, we tested the performance of each activity measure in terms of precision, recall, and F1. Activity measures for each detected player in a frame are computed based on motion features that are extracted within each player’s bounding box in that video frame in the case of DT+OF and DT+STIP and according to the confidence value of active player class detection in the case of DT+Y. The performance of all measures is evaluated on each frame by comparing the bounding box with the highest activity measure with the ground truth active player bounding box, as the DT+OF and DT+STIP measures are used in combination with the bounding boxes of detected players proposed by Yolo and DT+Y measure, as one of the outputs gives the bounding box of the active player. 

A detection was considered to be true positive if it was correctly labeled as active and had the largest IoU with the ground truth active player bounding box among the detected boxes. If the goal is to evaluate the performance of an activity measure, that is, how many times the measure has selected the right player as the most active, the exact match of the detected box with the ground truth is not crucial, but it is important to match GT as closely as possible (large IoU) if one wants to monitor player postures while performing an action. The minimum IoU overlap between the detected bounding box and ground truth was set to 10%, since, in this part, the focus was on the ability of the measure to distinguish between activity or inactivity of the player and not on the correct localization.

**Figure 13:**
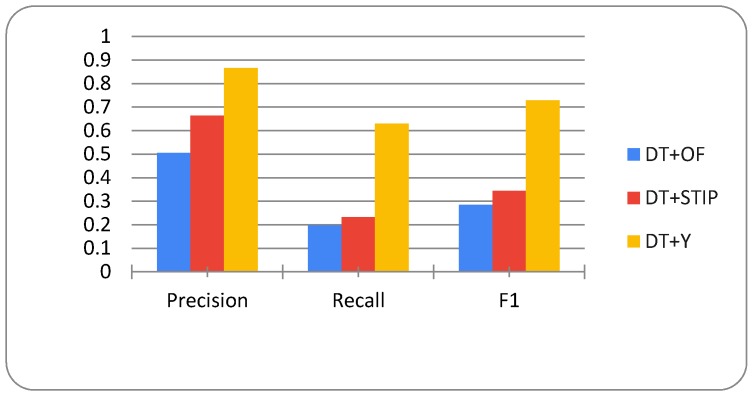
Evaluation results for DT+OF, DT+STIP, and DT+Y activity measures.

Figure 13 shows the evaluation results for all activity measures and indicates significantly higher values for the DT+Y activity measure. The DT+Y achieves the best F1 score of 73%, which is significantly better than the results that were achieved by other measures. DT+OF has a precision of 51%, a DT+STIP of 67%, and DT+Y of 87%. All of the measures achieve lower recall scores than precision scores, DT+OF achieves only 20%, DT+STIP 23%, and DT+Y 63%; however, this is an even more significant difference in DT+Y performance when compared to other measures.

**Figure 14:**
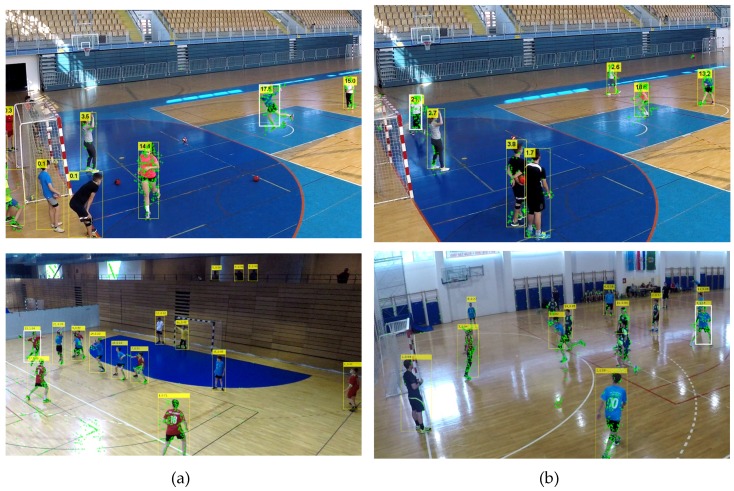
Marking of the leading player (white thick bounding box); (**a**) Correct detections; (**b**) Wrong detections. Examples were taken in different handball halls and under different indoor and outdoor lighting conditions.

Figure 14 shows examples of positive and negative detection of an active player. Negative detection occurred because the other player on the field had a greater activity measure than the player performing the required action who is supposed to be the most active.

It is necessary to know their activity measure in each frame, but also to uniquely identify that player throughout the period of interest, i.e., to track them through the sequence, to determine a player’s overall activity in a time period. The track of the active player $T_{b}=(B_{1},\;B_{1},\;\ldots .B_{b})$ on sequential frames is compared with the ground truth in order to evaluate the results of the proposed method for detecting an active player over a period of time.

A true positive rate was calculated as a number of true positives divided by the number of tested sequences to quantify the performance. An active player track is considered to be true positive if the IoU of the detected active player is equal to or greater than a threshold value α = 50% in more than θ of frames (here set to 50%). Formally, the measure for active player track evaluation is defined, as follows: 

Let be T a sequence of bounding box representations B, $T_{GT}$ a ground truth sequence, $IoU:TxT_{GT}\to \left[0,1\right]$ given function and f:T→{0,1}, being defined as:

(12) $$f\left(x\right)=\{\begin{array}{cc} \;1 & IoU\left(x,g\right)\geq \alpha \\ \;0 & IoU\left(x,g\right)<\alpha \end{array}\;$$

then a true positive ($TP_{T}$) for active player track T. is defined as:

(13) $$TPR_{T}=\{\begin{array}{cc} 1 & \frac{\sum_{B_{i}\in T}f\left(B_{i}\right)}{\left|T\right|}\geq \theta \\ 0 & \mathrm{else} \end{array}.\;$$

The $TP_{T}$ is computed for every track in a test set, so TPR% is the ratio of the true positive tracks to the total number of tracks in the test set.

This means that the measure is strictly defined, because only those results that have a true positive detection of the player in θ consecutive frames are considered to be positive, as long as the action is completed, with the additional condition that it should be the given handball action. Thus, the case when a player is detected and properly tracked during his movement, but does not perform an action of interest in more that θ frames is not counted as the true result. Additionally, when the player is correctly identified and followed only in the part of the sequence (less than θ) is not counted as a positive result.

Table 3 provides the evaluation results for active player detection method along the entire motion trajectory for all activity measures where IoU is set to α = 50% and at least θ = 50% of frames per track correctly detected. 

The best overall score was achieved with the DT+Y activity measure that was used in conjunction with the Deep Sort tracker with 67% correct active player sequences, while the second-best result was achieved using the DT+STIP activity measure and Deep Sort tracker, with 53% correct sequences. The gap in performance between the DT+STIP and DT+OF methods from the DT+Y is smaller here, where whole sequences are taken into account, than when being examined for their performance of activity detection in isolation. When considering whole sequences, if player detection and tracking performs well, some mislabeling of players as inactive in certain frames will not affect the label of the whole sequence, so the result will not degrade either. This is important to note, because even though the DT+Y activity measure performs the best for determining the activity of the player, to use it is necessary to have annotated training data, which, if not available, can be a time consuming and tedious job to acquire, and this is not required for the DT+OF and DT+STIP measures. An even larger set of learning data and data balancing is needed to achieve even better results of DT+Y measure, because the number of inactive athletes on the scene is much higher than the inactive ones.

With all activity measures, better results were obtained while using the Deep Sort tracker than with Hungarian algorithm-based tracking, so it seems a clear choice, since it does not require much more effort to use.

**Figure 15:**
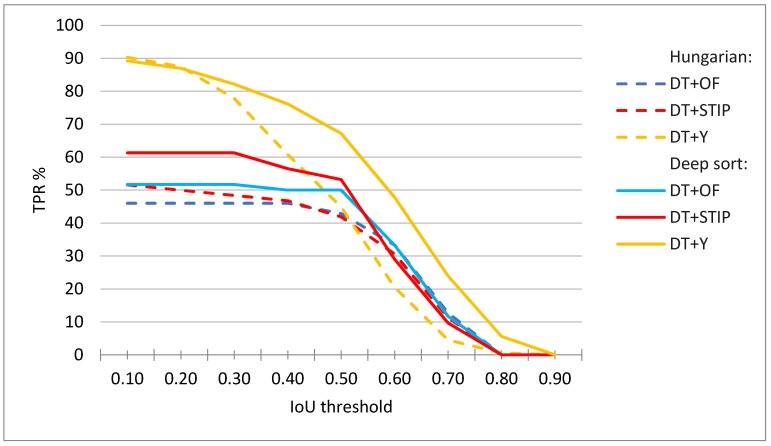
Evaluation results for player tracks obtained with DT+OF, DT+STIP, and DT+Y activity measures using Deep sort or Hungarian algorithm for tracking. The results at minimum θ = 50% correct frames per sequence.

We tested the proposed methods with different α threshold parameter and Figure 15 presents the results. In the case where only the information that the player is active for a certain period is relevant, the accuracy of detection is not as important (low overlap with the ground truth is acceptable) as in the case when we want to observe the player’s movements and postures when performing an action (large overlap with the ground truth is desirable). 

**Figure 16:**
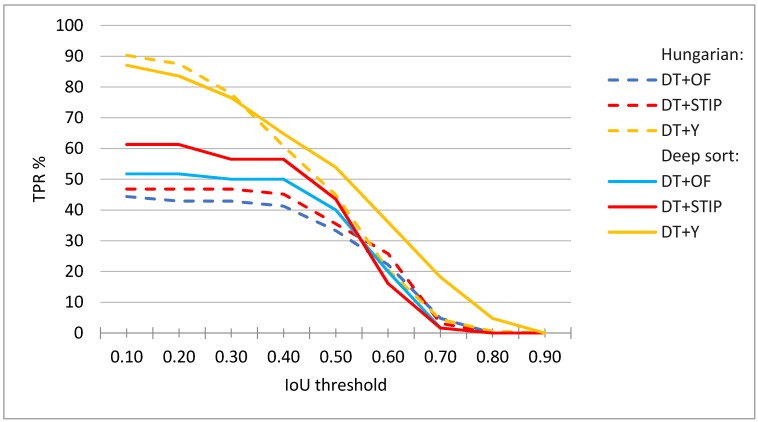
Evaluation results for player tracks obtained with DT+OF, DT+STIP, and DT+Y activity measures using Deep sort or Hungarian algorithm for tracking. The results at minimum θ = 70% correct frames per sequence.

Similar rankings hold for most values of the minimum required IoU (parameter α), as can be seen in Figure 16. 

When considering different values of minimum correct frames per sequence (parameter θ. ), again similar rankings can be observed, but, as expected, with different achieved TPR%. For example, Figure 16 shows that the TPR% is lower for various values of α when θ is increased to 70% than in the case shown in Figure 15. Here, the TPR score at lowest values of α is mostly limited by the performance of the tracking and activity detection, while at higher values of α. with the precision of player detection. 

**Figure 17:**
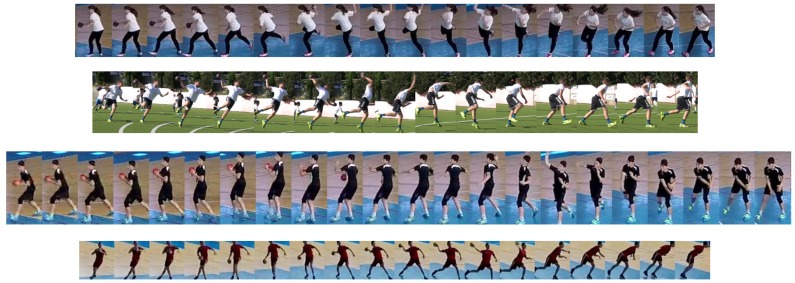
Examples of shooting action with and without jump performed by different players in different positions with respect to the camera in both outdoor and indoor fields.

In the following, some typical cases and issues are shown and commented on. Figure 17 shows a collage of thumbnails that present good examples of player tracks that include all correctly detected bounding boxes on the frames in the sequence. As each player performs a particular action in a specific way, the number of frames is variable, even when performing the same handball action or technique according to the same rules.

**Figure 18:**
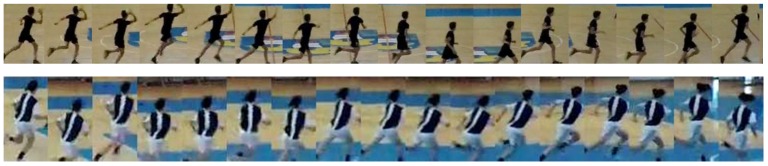
Examples of imprecise player detection.

**Figure 19:**
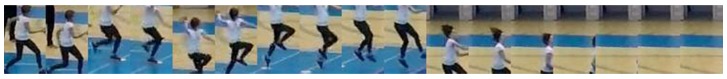
Tracking errors due to poor player detection.

The number of frames in player track depends on detector performance and tracking at all stages of the action. Figure 18 shows the problem of imprecise detection in some parts of the player tracking, where the detector has correctly detected the player, but the IoU is less than the threshold because his legs are outside of the bounding box. Figure 19 shows a negative example of an active player track that is caused by poor player detection that has propagated to other stages of the algorithm. The thumbnails show that the player is active and well tracked as long as it was detected. Additional training of the person class on samples from the handball domain can reduce the problem of imprecise detection.

**Figure 20:**
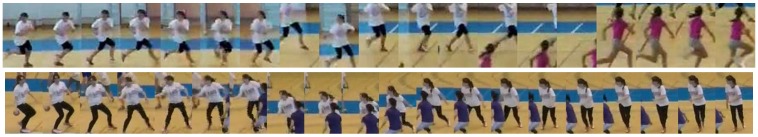
Examples of tracking problems.

Figure 20 shows a negative example of an active player’s track due to improper tracking of players occurring due to cluttered scenes, players overlapping, or changing position.

**Figure 21:**
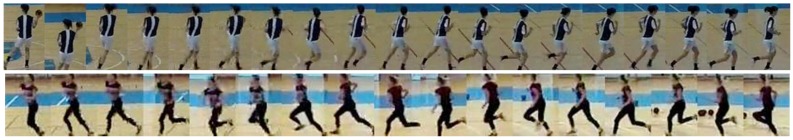
Tracking of players that perform actions outside of the scope.

A detector detects all players on each frame and the activity level is calculated for each one and their movement is monitored and recorded. The proposed method will select the player with the highest level of activity in most frames as the most active player; however, it is possible that the most active player is not the one performing the given action, so, in this case, there is no match to the GT, even though everything is done correctly. Figure 21 shows the cases when players who were preparing to perform an action or ran in the queue had a higher level of activity then player who was performing the default action and so were wrongly selected as active players.

As the two measures of activities DT + OF and DT + STIPS do not require any learning, we have tested them in completely different sports scenes from other team sports to test how general the proposed activity measures are.

We used the basketball event detection dataset [26] for the experiment. The dataset contains automatically obtained player detections and tracks for basketball events in 11 classes, such as three-point success, three-point failure, free-throw success, free-throw failure, and slam-dunk. Scenes were taken from a basketball game so that only one ball was present on the field. The clips are four seconds long and subsampled to 6fps from the original frame rate. The clips are tailored to include the entire event, from throwing the ball to the outcome of the shot. A subset of 850 clips also have the known ball location in the frame where the ball leaves the shooter’s hands. 

**Figure 22:**
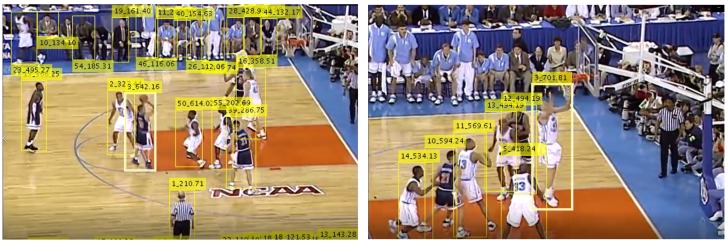
Marking of the leading player in basketball scenes (white thick bounding box).

We have used two activity measures, DT+OF and DT+STIPS, on the basketball event dataset without any modification. The DT+YOLO method was not tested, because it needs to be trained before use, and it was not trained on the basketball domain. The player detections and tracks provided with the dataset were used to evaluate only the impact of the activity measures on active player detection, Figure 22. 

In [26], the authors selected the player(s) who was closest in image space to the ball as the leading player ("shooter") to evaluate the attention mechanism. On the other hand, the handball scenes correspond to individual handball techniques and they were taken during training so that each player has his own ball, therefore the possession of the ball could not be taken as a measure of player activity and was not included in the design of proposed activity measures. For this reason, we selected a part of the basketball test set and determined the active player for the entire duration of the given action and then evaluated the active player detection metrics against these criteria.

The evaluation results for active basketball players detection along the entire motion trajectory for DT+OF and DT+SIFT activity measures, where at least θ = 50% of frames per track are correctly detected, as given in Table 4. 

The obtained results are lower than in the handball scenes (up to 8%), primarily because the video sequences in basketball dataset are event-driven rather than determined by the action for which the method was designed. This is most noticeable on actions, such as three-point throw, where it happens that the player who threw the ball is not even present in most of the frames as the camera zooms in on the ball’s trajectory and the surrounding players and basket to track the outcome of the throw. Contrary to the method of detecting events in the basketball game, the proposed methods are defined from the perspective of the player and they correspond to some handball techniques, for example, a shot jump takes from the moment the player receives the ball, takes up to three steps, and throws the ball towards the goal, regardless of the outcome of the action (the ball enters/does not enter the goal).

The results are significant and promising, because they are comparable and even slightly better than the results that were reported in [26], although the method in [26] was purposefully trained to detect an active player on the basketball dataset while using 20 GPUs. 

The track results exhibit similar properties as on the handball dataset, with problems stemming from the same sources—player detection, tracking, or activity measure. The difference is that basketball has fewer players on the field, smaller terrain, bigger ball, and the player’s occlusion in basketball is even greater than in handball scenes because of the speed and difference in the rules of the game. Additionally, in our handball dataset, the actions are defined from the player’s perspective and they correspond to individual handball techniques. 

**Figure 23:**
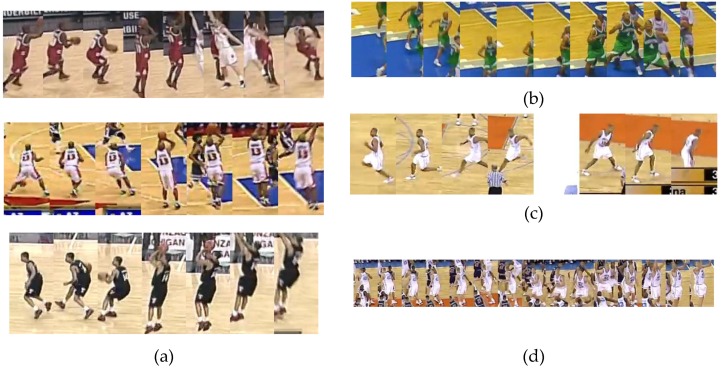
Example of results on the basketball dataset. (**a**) Correct tracks for the three-point failure event; (**b**) Wrong result due to imprecise player detection; (**c**) Incorrect result due to higher activity of player who is not performing the action of interest; (**d**) Incorrect results due to bias to longer tracks.

Finally, Figure 23 presents a visual sample of results on the basketball set. 

For this reason, for the final track selection, our method assumes that the player should be tracked throughout the whole video sequence, so it is, by design, biased towards longer tracks. It often misses choosing the shooter in cases, such as three-point shot, where the player who throws the ball might not even be present in the majority of the clips (Figure 23d). Track fragmentation, due to poor tracking, where the player that should be labeled as active is actually labeled with more than one track ID, impairs the ability to select the correct track for the same reason. The wrong selection of active track can happen even though the correct player might have been the most active in the frame the ball leaves the shooter’s hands. A similar bias toward longer tracks is reported in [26] for the tracking-based attention model. In future work, the method should be able to switch the leading player track from player to player to handle this problem and extend the application to longer, temporally unsegmented clips.

## 5. Conclusions

There is great interest in the automatic interpretation of sports videos, for the purposes of analysis of game tactics and individual athlete’s performance, for retrieval, sports analysis and monitoring and refinement of player action techniques and playing style. To facilitate automatic analysis in team sports, such as handball, the detection and tracking of players who are currently performing a relevant action is of particular interest, as the interpretation of the whole scene often depends on that player’s action. Additionally, during training sessions, the time should be efficiently utilized, so that all players are active in sufficient proportion. 

In this paper, an active player detection method is proposed, which entails frame-by-frame object detection, player activity measures, and tracking methods to detect and track active players in video sequences. The CNN-based Yolo object detector was used for player detection, Hungarian assignment algorithm based method, and Deep SORT were considered for tracking the detected players along the time dimension, and three different measures are proposed for the task of player activity detection in a certain frame, DT+OF, DT+STIP, and DT+Y. The DT+STIP measure is calculated from the spatiotemporal interest points (STIPs) that were detected within the area of a player, the simpler DT+OF method depends on the computed optical flow (OF) motion field and the DT+Y method uses convolutional neural network (CNN) for determining player activity and classifying active and inactive players.

The proposed method and its components were tested on the test set of handball videos from several training sessions that were acquired in the wild. A sequence-based evaluation measure was proposed for overall evaluation, and the best result was achieved with the Deep Sort tracking method with the CNN-based DT+Y activity measure, achieving the rate of 67% correct sequences on the test set. The DT+Y method requires training data, and if it is not available, the DT+STIP or DT+OF method can be used, which achieved 53% TPR and 50% TPR on the test set, respectively.

To test how general the proposed activity measures are, we have chosen completely different sport scenes from basketball sports domain and evaluated the performance of DT+OF and DT+STIPS measures, since they do not require any leaning. The obtained results are lower than in the handball scenes (up to 8%), primarily because the video sequences are event-driven rather than determined by the action, as in our case, and there is an even greater occlusion of the players, as basketball is played faster and on a smaller field. The results are promising, because they are comparable to the results of a method that was trained on that basketball dataset to detect an active player in a video sequence.

The achieved results confirm that active player detection is a very challenging task and it could be further improved by improving all three components (player detection, tracking, and activity measure). Particularly, player detection should be improved first, because both other components directly depend on its performance. Activity measure should be calculated inside the detected bounding box, so imprecise detection will negatively affect this measure, since parts of player body may be outside the detected box and irrelevant parts of background within. Similarly, in addition to the inability to track objects that are not detected at all, the Deep Sort tracking method uses visual history of previous appearances of players, so their imprecise detection impacts the ability of the tracker to correctly re-identify them after occlusion. 

For application in match setting, where a single ball exists in play, ball detection could be incorporated as an additional significant clue for detecting the leading player. It is indisputable that the information that the ball has in the game is important for determining the action and the player, but it is a small object that is hidden in the player’s hand most of the time, and, when thrown quickly, moves and changes direction, so it is often blurred and tracking is still a challenge that is being intensively tried to tackle.

In the future, the method should be extended to better deal with multi-player actions, such as crossing or defense, and the player activity could be monitored in relation to the type of action the player is performing. Additionally, the method should be extended to handle long-term video clips, where the leading player changes in different time intervals. 

## Figures and Tables

**Table 1:** The track score vectors for each detected player on video sequence is presented in Figure 7. Number of wins for each player in a sequence and final rankings are highlighted.

	Player ID	F1 Ranking	F2 Ranking	F3 Ranking	F4 Ranking	F5 Ranking	Number of Wins	Final Ranking
**Track score vectors**	1	2	2				**1**	**0**
	2	3	3	2	3		**0**	**0**
	6	1	1	1	1	1	**5**	**1**
	9				2	2	**0**	**0**

**Table 2:** Number of detected players per frame.

	Min	Mean	Max	Median
No. players	2	11.55	26	11

**Table 3:** True positive rates (TPR %) for active player detection.

	TPR % for Activity Measures		
Tracking	DT+OF	DT+ STIP	DT+Y
Hungarian	42.9	41.9	32.8
Deep Sort	50.0	53.2	67.2

**Table 4:** True positive rates for active player detection in basketball event detection scenes.

Activity Measure	DT+OF	DT+ STIP	Shooter Detection [26]
TPR %	43.87	45.26	43.8

## References

[1] Buric M., Pobar M., Ivasic-Kos M. An overview of action recognition in videos. Proceedings of the 40th International Convention on Information and Communication Technology, Electronics and Microelectronics (MIPRO).

[2] Redmon J., Farhadi A. (2018). Yolov3: An incremental improvement. arXiv.

[3] Wojke N., Bewley A., Paulus D. Simple online and realtime tracking with a deep association metric. Proceedings of the 2017 IEEE International Conference on Image Processing (ICIP).

[4] Kristan M., Leonardis A., Matas J., Felsberg M., Pflugfelder R., Cehovin Zajc L., Vojir T., Hager G., Lukezic A., Eldesokey A. The visual object tracking vot2017 challenge results. Proceedings of the IEEE International Conference on Computer Vision Workshops.

[5] Dendorfer P., Rezatofighi H., Milan A., Shi J., Cremers D., Reid I., Roth S., Schindler K., Leal-Taixe L. (2019). CVPR19 Tracking and Detection Challenge: How crowded can it get?. arXiv.

[6] Okuma K., Taleghani A., De Freitas N., Little J.J., Lowe D.G. (2004). A boosted Particle Filter: Multitarget Detection and Tracking. European Conference on Computer VISION.

[7] Pers J., Kovacic S. Computer vision system for tracking players in sports games. Proceedings of the First International Workshop on Image and Signal Processing and Analysis. Conjunction with 22nd International Conference on Information Technology Interfaces, IWISPA 2000.

[8] Needham C.J., Roger D.B. (2001). Tracking multiple sports players through occlusion, congestion and scale. BMVC.

[9] Erikson M., Ferreira A., Cunha S.A., Barros R.M.L., Rocha A., Goldenstein S. (2014). A multiple camera methodology for automatic localization and tracking of futsal players. Pattern Recognit. Lett..

[10] Bebie T., Bieri H. SoccerMan-reconstructing soccer games from video sequences. Proceedings of the 1998 International Conference on Image Processing. ICIP98 (Cat. No.98CB36269).

[11] Xu M., Orwell J., Jones G. Tracking football players with multiple cameras. Proceedings of the 2004 International Conference on Image Processing, ICIP’04.

[12] Jia L., Tong X., Li W., Wang T., Zhang Y., Wang H. (2009). Automatic player detection, labeling and tracking in broadcast soccer video. Pattern Recognit. Lett..

[13] Zhu G., Xu C., Huang Q., Gao W. Automatic multi-player detection and tracking in broadcast sports video using support vector machine and particle filter. Proceedings of the 2006 IEEE International Conference on Multimedia and Expo.

[14] Lehuger A., Duffner S., Garcia C. (2007). A Robust Method for Automatic Player Detection in Sport Videos.

[15] Bewley A., Ge Z., Ott L., Ramos F., Upcroft B. Simple online and realtime tracking. Proceedings of the 2016 IEEE International Conference on Image Processing (ICIP).

[16] Pobar M., Ivašić-Kos M. (2019). Detection of the leading player in handball scenes using Mask R-CNN and STIPS. Proceedings of the Eleventh International Conference on Machine Vision (ICMV 2018).

[17] Ali B., Itti L. (2012). State-of-the-art in visual attention modeling. IEEE Trans. Pattern Anal. Mach. Intell..

[18] Laurent I., Koch C., Niebur E. (1998). A model of saliency-based visual attention for rapid scene analysis. IEEE Trans. Pattern Anal. Mach. Intell..

[19] Junwei H., Zhang D., Cheng G., Liu N., Xu D. (2018). Advanced deep-learning techniques for salient and category-specific object detection: A survey. IEEE Signal Process. Mag..

[20] Marat S., Phuoc T.H., Granjon L., Guyader N., Pellerin D., Guérin-Dugué A. (2009). Modelling spatio-temporal saliency to predict gaze direction for short videos. Int. J. Comput. Vis..

[21] Pobar M., Ivasic-Kos M. Mask R-CNN and Optical flow based method for detection and marking of handball actions. Proceedings of the 11th International Congress on Image and Signal Processing, BioMedical Engineering and Informatics (CISP-BMEI).

[22] Mihir J., Jan van G., Jégou H., Bouthemy P., Snoek C.G.M. Action localization with tubelets from motion. Proceedings of the IEEE Conference on Computer Vision and Pattern Recognition.

[23] Georgia G., Malik J. Finding action tubes. Proceedings of the IEEE Conference on Computer Vision and Pattern Recognition.

[24] Alexander K., Marszałek M., Schmid C., Zisserman A. (2010). Human focused action localization in video. Proceedings of the European Conference on Computer Vision.

[25] Dalal N., Triggs B. Histograms of oriented gradients for human detection. Proceedings of the IEEE Computer Society Conference on Computer Vision and Pattern Recognition (CVPR’05).

[26] Ramanathan V., Huang J., Abu-El-Haija S., Gorban A., Murphy K., Fei-Fei L. Detecting events and key actors in multi-person videos. Proceedings of the IEEE Conference on Computer Vision and Pattern Recognition.

[27] Ivasic-Kos M., Iosifidis A., Tefas A., Pitas I. Person de-identification in activity videos. Proceedings of the 37th International Convention on Information and Communication Technology, Electronics and Microelectronics (MIPRO).

[28] Buric M., Pobar M., Ivašić-Kos M.I. Object Detection in Sports Videos. Proceedings of the 41st International Convention on Information and Communication Technology, Electronics and Microelectronics (MIPRO).

[29] Burić M., Pobar M., Ivašić-Kos M. Ball detection using YOLO and Mask R-CNN. Proceedings of the International Conference on Computational Science and Computational Intelligence (CSCI).

[30] He K., Gkioxari G., Dollár P., Girshick R. Mask r-cnn. Proceedings of the IEEE International Conference on Computer Vision.

[31] Burić M., Pobar M., Ivašić-Kos M. Adapting YOLO network for Ball and Player Detection. Proceedings of the 8th International Conference on Pattern Recognition Applications and Methods (ICPRAM 2019).

[32] Redmon J., Divvala S., Girshick R., Farhadi A. You only look once: Unified, real-time object detection. Proceedings of the IEEE Conference on Computer Vision and Pattern Recognition.

[33] Munkres J. (1957). Algorithms for the assignment and transportation problems. J. Soc. Ind. Appl. Math..

[34] Barron J.L., Fleet D.J., Beauchemin S.S. (1994). Performance of optical flow techniques. Int. J. Comput. Vis..

[35] Laptev I. (2005). On space-time interest points. Int. J. Comput. Vis..

[36] Dollár P., Rabaud V., Cottrell G., Belongie S. Behavior recognition via sparse spatio-temporal features. Proceedings of the IEEE International Workshop on Visual Surveillance and Performance Evaluation of Tracking and Surveillance.

[37] Klaser A., Marszalek M., Schmid C. A Spatio-Temporal Descriptor Based on 3D-Gradients. Proceedings of the BMVC 2008-19th British Machine Vision Conference, British Machine Vision Association.

[38] Chakraborty B., Holte M.B., Moeslund T.B., Gonzàlez J. (2012). Selective spatio-temporal interest points. Comput. Vis. Image Underst..

[39] Ivasic-Kos M., Pobar M., Ribaric S. (2016). Two-tier image annotation model based on a multi-label classifier and fuzzy-knowledge representation scheme. Pattern Recognit..

